# Targeting Intrinsically Disordered Proteins through Dynamic Interactions

**DOI:** 10.3390/biom10050743

**Published:** 2020-05-11

**Authors:** Jianlin Chen, Xiaorong Liu, Jianhan Chen

**Affiliations:** 1 Department of Hematology, Taizhou Central Hospital (Taizhou University Hospital), Taizhou 318000, Zhejiang, China; jianlinchen79@yahoo.com; 2 Department of Chemistry, University of Massachusetts, Amherst, MA 01003, USA; xiaorongliu@umass.edu; 3 Department of Biochemistry and Molecular Biology, University of Massachusetts, Amherst, MA 01003, USA

**Keywords:** aggregation, cancer, disordered ensemble, drug design, enhanced sampling, GPU computing, molecular dynamics, neurodegenerative diseases, p53, protein force fields

## Abstract

Intrinsically disordered proteins (IDPs) are over-represented in major disease pathways and have attracted significant interest in understanding if and how they may be targeted using small molecules for therapeutic purposes. While most existing studies have focused on extending the traditional structure-centric drug design strategies and emphasized exploring pre-existing structure features of IDPs for specific binding, several examples have also emerged to suggest that small molecules could achieve specificity in binding IDPs and affect their function through dynamic and transient interactions. These dynamic interactions can modulate the disordered conformational ensemble and often lead to modest compaction to shield functionally important interaction sites. Much work remains to be done on further elucidation of the molecular basis of the dynamic small molecule–IDP interaction and determining how it can be exploited for targeting IDPs in practice. These efforts will rely critically on an integrated experimental and computational framework for disordered protein ensemble characterization. In particular, exciting advances have been made in recent years in enhanced sampling techniques, Graphic Processing Unit (GPU)-computing, and protein force field optimization, which have now allowed rigorous physics-based atomistic simulations to generate reliable structure ensembles for nontrivial IDPs of modest sizes. Such de novo atomistic simulations will play crucial roles in exploring the exciting opportunity of targeting IDPs through dynamic interactions.

## 1. Introduction

Proteins are central components of regulatory networks that dictate virtually all aspects of cellular decision-making [1]. Demand for more sophisticated signaling in complex multicellular organisms has been met with increasing utilization of proteins that are highly flexible [2,3,4]. In particular, so-called intrinsically disordered proteins (IDPs) account for ~50% of signaling-associated proteins in eukaryotes [5]. These proteins have lower sequence complexity compared to folded proteins, lacking large hydrophobic residues and enriched with charged and polar ones [6]. They do not have stable tertiary structures in the unbound state under physiological conditions, even though they frequently undergo folding transitions upon binding to specific targets [7]. The inherent thermodynamic instability of the structural features of this class of proteins allows their conformational properties to respond sensitively to numerous stimuli, including the binding of various small and large molecules, changes in cellular environments (e.g., pH), and post-translational modifications [8,9,10,11,12,13]. Multiple signals could also be naturally integrated through cooperative responses of the dynamic structure ensemble (such as coupled binding and folding) [14]. These properties make IDPs uniquely suitable for fulfilling the complex signaling need of higher organisms. At the same time, deregulation of IDPs has been associated with many human diseases, including cancers, neurodegenerative diseases, heart disease, and diabetes [5,15,16,17,18,19,20]. For example, over two-thirds of cancer-associated proteins have been predicted to contain extensive regions of intrinsic disorder [5], and predicted disordered regions have been estimated to house almost one quarter of disease-associated missense mutations [21]. There is thus tremendous interest in determining if and how IDPs may be targeted for therapeutic purposes. 

The dynamic and heterogeneous nature of unbound IDPs presents substantial challenges for characterization and this has proven to be a major bottleneck for establishing a reliable sequence–structure–function–disease relationship of IDPs [14,22,23,24,25,26]. The lack of a clear understanding of the molecular basis of IDP function and deregulation in diseases has created significant ambiguity on the druggability of most IDPs, including transcription factors [16]. Most existing case studies of targeting IDPs have focused on extending the traditional structure-based screening and drug design strategies and emphasize exploiting residual structures and pre-existing potential binding pockets of the unbound state [27,28,29,30,31,32,33,34,35,36,37,38,39,40,41,42,43]. Nonetheless, it is clear that the disordered nature of IDPs would require novel strategies for targeting as well as new conceptual frameworks for thinking about how small molecule binding could modulate IDP structure and function. In particular, it has been recognized that it may be more useful to consider the problem of targeting IDPs in the context of structural ensemble modulation [44], even though it is generally believed that one still needs to achieve specific interactions, such as by exploiting pre-existing structural features [45]. Many outstanding reviews have already been dedicated towards existing examples along these lines and they also provide extensive discussion of the successes, opportunities, and challenges of targeting IDPs via specific interactions of small molecules in neurodegenerative diseases, cancers, and other diseases [18,45,46,47,48,49,50,51,52,53,54,55]. 

In this review, we will first summarize important recent advances in physics-based de novo simulations of disordered protein ensembles, including Graphic Processing Unit (GPU) computing, enhanced sampling, and re-balanced protein force fields, and then focus on emerging examples that suggest the exciting possibility of targeting IDPs by directly modulating the disordered ensembles through dynamic and transient interactions. We will discuss the promise of such a broader view of how IDPs may be targeted as well as key challenges and required methodological developments to support targeting IDPs via dynamic interactions.

## 2. Characterization of Disordered Protein Ensembles: A Crucial Role for Atomistic Simulations

A principal challenge in understanding the druggability and best targeting strategy of IDPs resides in the difficulty of detailed characterization of disordered protein states [14,23,24,56]. These states need to be represented using heterogeneous structure ensembles and are not amenable to traditional high-resolution structure determination methods. Here, we briefly discuss the current status and challenges of disordered protein ensemble determination, which has a direct impact on the ability to devise effective strategies of designing IDP binders and optimizing leads identified from traditional screening efforts.

### 2.1. Fundamental Challenges in Experimental Determination of Disordered Protein Ensembles

Experimentally, a wide range of biophysical methods can be applied to characterize disordered protein states, including NMR, circular dichroism (CD), small-angle X-ray scattering (SAXS), Förster resonance energy transfer (FRET), hydrogen/deuterium (H/D) exchange, mass spectrometry, and others [57,58]. These methods can provide complementary information on the local, intermediate, and long-range structural organizations of IDPs. NMR in particular is arguably the most powerful technique for structural studies of IDP. Many NMR observables can be measured at residue and atomic levels to infer secondary and tertiary structural properties. SAXS and FRET are highly complementary to NMR and provide information on the long-range global organization of the disordered ensemble. Yet, a fundamental limitation is that these experimental measurements generally reflects the average properties, which alone are not sufficient to uniquely define the underlying heterogeneous ensemble due to the severely underdetermined nature of the structural calculation problem [22,23,25,59,60,61]. At present, the most robust methods generally involve first generating a large number of candidate random structures and then using experimental structural restraints to select and construct optimal sub-ensembles according to various statistical criteria [62,63,64,65,66,67,68,69,70,71]. Nonetheless, these methods rely critically on the ability to generate initial candidate structures that are not only diverse enough to cover the range of accessible states of the protein but also specific enough to contain any nontrivial local (and long-range) structure features associated with a particular protein state. These two requirements are difficult to satisfy simultaneously for most proteins of moderate sizes (e.g., 50–100 residues or longer) and complexity. As a consequence, disordered ensembles constructed using these approaches depend critically on the underlying protein model and/or coil library. Furthermore, these ensembles are generally not proper thermodynamic ensembles. They should be considered just as ensemble models and cannot be used to reliably quantify statistical properties and extract thermodynamic parameters. As such, these experimental restraint-based ensemble construction approaches are likely inadequate for capturing potentially subtle effects of ligand binding on the disordered ensemble. 

### 2.2. Recent Advances in de Novo Simulations of Disordered Protein Ensembles

Given the fundamental challenges of disordered ensemble modeling based on experimental restraints alone, physics-based atomistic simulations have a crucial role to play in helping elucidate the conformational properties of IDPs and establishing a reliable molecular basis of their function and regulation [72,73,74,75,76]. A particularly attractive approach is to first generate the atomistic ensemble using a transferable physics-based force field in absence of any experimental restraints and then use the experimental data for independent evaluation of the quality of the simulated ensemble. Such a de novo simulation approach can effectively overcome the under-determined nature of disordered ensemble calculation, by leveraging the laws of physics that govern the nature of conformational fluctuation of the protein. Successful simulations of disordered protein ensembles have proven to be very challenging, requiring both accurate description of the conformational dependence of energy and sufficient sampling of relevant conformational space of the protein. Early IDP simulations suffered from both systematic biases in the general-purpose protein force fields and severe limitations in conformational sampling [14]. Nonetheless, these physics-based simulation approaches promise to provide rigorous thermodynamic ensembles required for reliable description of IDP–ligand interactions. The accuracy and capability of de novo IDP simulations can be expected to improve continuously over time, benefiting from robust advances in molecular simulation methodologies and high-performance computing hardware. Indeed, important breakthroughs have been made in the last few years in force field accuracy and sampling capability, which arguably have now allowed reliable de novo simulations of at least moderate-sized IDPs in general.

#### 2.2.1. Overcoming Sampling Bottleneck using Enhanced Sampling and GPU Computing

One of the most important recent advances in molecular dynamics (MD) simulations is the widespread availability of efficient GPU-enabled algorithms in virtually all major molecular simulation packages [77,78,79,80,81,82]. Modern GPUs can process thousands of threads in parallel to accelerate explicit solvent MD simulations by up to 100× compared to traditional CPU computing, significantly boosting the sampling capability. For example, the most efficient GPU-enabled MD codes can yield 100–200 ns per day for systems of ~1,000,000 atoms on a single NVIDIA RTX 2080Ti GPU card that costs only ~$1000. The ability to efficiently sample the protein conformational space has further benefitted from the emergence of enhanced sampling techniques [83,84,85,86,87,88,89,90], particularly various replica exchange (REX)-based methods. Among the various REX methods, replica exchange with solute tempering methods (REST) [86,91] is particularly suitable for atomistic simulations of IDPs in explicit solvent. In REST, a selected region of the system (e.g., the protein solute or a flexible segment of the protein) is subjected to tempering (i.e., random walk in the temperature space) while the rest of the system is maintained at a constant temperature (e.g., room temperature). This is achieved by scaling the interactions within the selected region and between it and the rest of the system. Because the number of required replicas of replica exchange simulations scales as the square root of the number of atoms, REST can significantly reduce the number of replicas required for covering the needed temperature space (by ~3-fold), thus overcoming a critical drawback in traditional temperature REX simulation in explicit solvent. The role of enhanced sampling in the recent successes of simulating dynamic IDP–ligand interactions will become evident in the later sections of this review.

**Figure 1:**
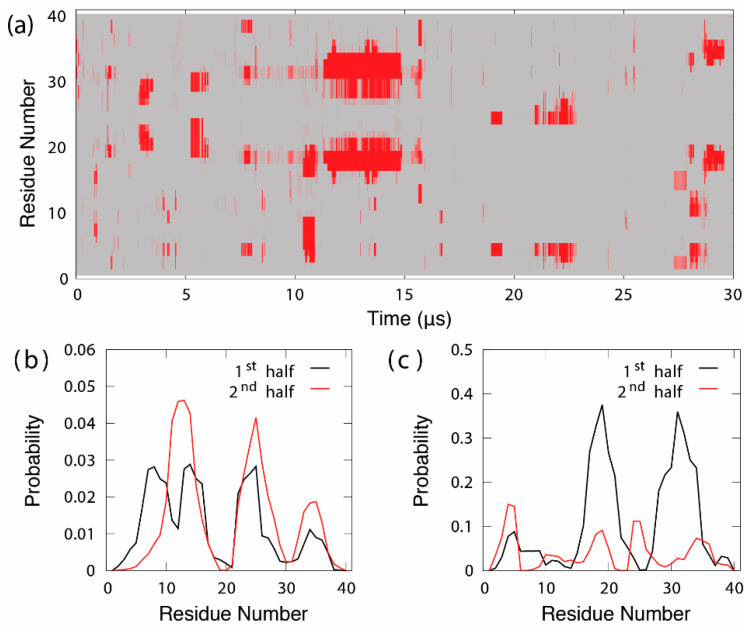
(**a**). Evolution of the per-residue β-sheet structure during a 30-μs Anton MD simulation of intrinsically disordered Aβ40 in explicit solvent at 300 K. Residues assigned to be in the β-sheet conformation are colored in red. (**b**) Average residue helix and (**c**) β-sheet probability profiles derived from the first and second halves of the trajectory. Note that both pairs of profiles differ significantly, reflecting a lack of convergence in the simulated disordered ensemble. The original MD trajectory was generously provided by D. E. Shaw Research [92].

The importance of enhanced sampling for the generation of converged ensembles cannot be over emphasized. For example, Figure 1A shows the evolution of the per-residue β-sheet structure during a 30-μs conventional MD simulation of an intrinsically disordered Aβ40 peptide in explicit solvent at 300 K, performed with Anton specialized hardware using the well optimized a99SB-disp force field [92]. Note that this trajectory is one to two orders of magnitude longer than typical MD simulations performed using general purpose CPU- or GPU-based high-performance computing platforms. Yet, very few reversible transitions are observed. For example, a transient β-hairpin spanning residues 15 to 36 persists for about 5 μs from ~10 to 15 μs and never appears again for the rest of the simulation. As a result, the final average residue helix and β-sheet probability profiles calculated from the first and second halves of the trajectory differ greatly, reflecting a very limited level of convergence. There is thus danger in relying on standard MD simulations in deriving quantitative characterizations of disordered protein ensembles. It should also be emphasized that achieving a sufficient level of convergence required for resolving potentially subtle changes in the disordered ensemble, such as upon ligand binding, can be extremely challenging even with enhanced sampling. It is critical to carefully analyze and establish the level of convergence for proper interpretation of simulated ensembles. Ideally, one should perform two or more independent simulations using distinct initial conformations and compare the resulting ensembles. The simulated ensemble from a single continuous run may appear to stop changing with respect to simulation time due to trapping in numerous local energy minima, giving rise to a misleading impression of convergence.

#### 2.2.2. Balanced Explicit Protein Force Fields for Describing Disordered Protein Ensembles

The dramatically improved sampling capability has facilitated extensive efforts to reparametrize general-purpose protein force fields to achieve greater balance of describing protein conformational equilibria. Studies of disordered protein states have been a key driver of these developments and several well-characterized IDPs have been widely used as training systems and/or benchmarks for force field optimization [92,93,94,95,96,97,98,99]. Many force field variants have been developed in recent years, including Amber ff98SB [100], ff99SB*-ILDN [101] and variants [95,102], ff03ws [103], ff14SB [104], ff99SBnmr [105], CHARMM22* [106], CHARMM36m (and C36mw) [93], a99SB-disp [92], and others. A key focus of these optimization efforts has been to rebalance the protein–protein, protein–water, and water–water interactions. Earlier versions of general-purpose protein force fields consistently over-stabilize nonspecific protein–protein interactions and lead to overly compact conformational ensembles for disordered protein states [107,108]. It was demonstrated that such bias could be effectively compensated by directly increasing the strengths of protein–water dispersion interactions [95,103,109], even though other components of the force field should also be reparametrized for self-consistency. The latest CHARMM36m and a99SB-disp force fields, in particular, have been systematically reparametrized based on extensive simulations of tens of globular and disordered proteins and achieve impressive levels of accuracy for describing both structured and unstructured proteins.

**Figure 2:**
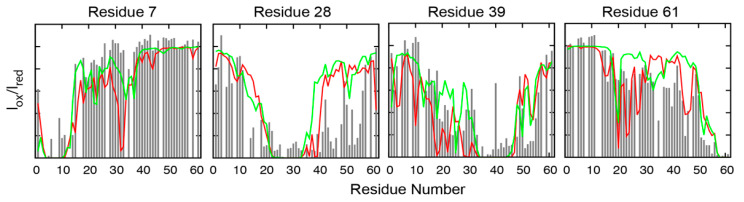
Calculated (lines) and experimental (grey bars) NMR Paramagnetic Relaxation Enhancement (PRE) effects induced by paramagnetic spin labelling at residues D7, E28, A39, and D61 of p53-TAD. Red and green traces were calculated from an independent control and folding REST2 simulations of p53-TAD in a99SB-disp, respectively, to evaluate the level of convergence. Control and folding simulations were initiated from helical and fully unstructured structures, respectively, and the length of REST2 simulations were 1 μs per replica. This figure was adapted from [99]. See [99] for details on the simulation and analysis.

In a recent benchmark study, six of the latest protein force fields were evaluated using the 61-residue N-terminal transactivation domain (TAD) of tumor suppressor p53, which is a very challenging system due to its size and complex conformational features [99]. It has been extensively characterized by NMR, SAXS, and single-molecule FRET and shown to contain a range of nontrivial local and long-range residual structures [110]. The disordered ensemble of p53-TAD was calculated using REST2-enhanced sampling using GPU-accelerated GROMACS 5.1.4 [80,111] patched with PLUMED 2.3.0 [112,113,114]. Each REST2 simulation lasted 1.0 μs per replica, representing one of the most extensive atomistic simulations of IDPs of similar sizes. The results show that the ensembles generated using the force field a99SB-disp yield the best agreement with the experimental data at both secondary and tertiary structure levels. For example, the back-calculated NMR paramagnetic relaxation enhancement (PRE) effects are highly consistent with the experimental results for all four available labelling sites (Figure 2). This suggests that the simulated ensembles not only have the proper overall chain dimension but also recapitulate much of the transient long-range ordering within the unbound state of p53-TAD. The latter is an extremely challenging task. The fact that this could be achieved by a99SB-disp represents an exciting breakthrough, suggesting that de novo atomistic simulations are now ready to provide a reliable approach for detailed characterization of the disordered ensembles of at least moderately sized IDPs.

#### 2.2.3. Implicit Solvent as a Promising Alternative for Simulating Disordered Ensembles

We note that implicit solvent protein force fields have also been developed and deployed for atomistic simulations of IDPs with various levels of success [75,96,98,115]. Implicit treatment of solvent reduces the simulation system size ~10-fold by direct estimation of the solvation free energy. It could provide important advantages for satisfying the simultaneous requirements of adequate sampling and sufficient force field accuracy for simulating disordered protein states. The ABSINTH model, in particular, has demonstrated significant successes in mapping the sequence–conformational space relationship of IDPs [25]. An improved version named ABSINTH-C was recently developed by including the backbone torsion cross-terms optimized based on experimentally derived statistics [98]. Independently, the generalized Born with the molecular volume 2 (GMMV2) model, which is considered one of the most accurate implicit solvent models, was recently implemented on GPU within the CHARMM/OpenMM interface [116]. GPU-accelerated GBMV2 is about 60-fold faster and provides a competitive alternative to explicit solvent simulations for studying the IDP structure and interaction. This model was previously optimized based on enhanced sampling of model peptides and shown to be capable of accurately describing the conformational properties of both folded and unfolded peptides [96]. The development of the GPU-accelerated version thus removed a key bottleneck to broader application of GBMV2 for atomistic simulations of the IDP structure and interactions. Nonetheless, whether these implicit solvent models could provide a viable alternative to explicit solvent simulations in studies of IDP–ligand interactions is yet to be demonstrated.

## 3. Modulating Disordered Protein Ensembles via Dynamic Interactions

To date, small molecular targeting of IDPs has mostly focused on proteins involved in neurodegenerative diseases, such as amyloid β (Aβ) peptides, α-synuclein, and tau protein, and disordered regions of cancer-associated transcription factors, such as p53, c-Myc, EWS-FLI1, KLF5, and others [16,117]. Advances in experimental and computational methods for studying disordered protein ensembles have allowed examination in greater details the interactions between IDPs and small molecules. There has been an emergence of examples showing that small molecules could modulate the disordered ensemble itself through nonspecific and dynamic interactions and achieve specific functional effects, in complete contrast to the traditional paradigm of drug binding that emphasizes strong specific interactions. Such a dynamic mode of IDP–small molecule interactions is reminiscent of “fuzzy complexes” in protein–protein interactions involving IDPs [118,119,120]. The observation that small molecules could induce substantial effects through dynamic interactions is fascinating and suggests a broader and more effective strategy for targeting IDPs in general. 

### 3.1. Dynamic Interactions of c-Myc Inhibitors

One of the earliest examples of dynamic interactions between small molecules and IDPs concerns c-Myc inhibitors [27,28]. NMR, CD, and fluorescence studies initially suggested that these inhibitors bound specifically to multiple independent sites in the monomeric and disordered c-Myc, which induced Max-binding-incompatible conformations to disrupt the c-Myc–Max interaction [27]. Subsequent explicit solvent simulations by Michel and Cuchillo in 2012 [121] revealed that the interaction between c-Myc and one of the inhibitors, 10058-F4, was actually dynamic and involved many short-lived contacts. Such a dynamic and nonspecific nature of the interaction could explain the observation that small modifications to the ligand had limited effects on the binding affinity [122]. A similar mode of interaction was proposed by Jin et al. in 2013 [123] for another c-Myc inhibitor, 10074-A4, where atomistic simulations in explicit and implicit solvent force fields revealed the ligand to form a “ligand cloud” and interact dynamically with the disordered “protein cloud”. Importantly, the dynamic nature of the interaction between 10058-F4 and c-Myc_402-412_ can confer sequence specificity, even though the interaction involves many transient contacts instead of well-defined specific ones [124]. It has been further suggested that such dynamic interactions may be driven by entropic expansion of the IDP conformational space [51]. Indeed, thermodynamics analysis showed that binding of 10058-F4 to c-Myc_402-412_ was dominated by the entropic contribution (−20.7± −4.2 kJ/mol out of the total binding free energy of −27.6 ± −8.5 kJ/mol) [124]. The caveat, however, is that binding of hydrophobic ligands is generally associated with large entropic contributions due to the release of restricted water molecules near the hydrophobic surface. It is thus not clear if c-Myc indeed undergoes entropic expansion upon 10058-F4 binding. An in-depth NMR study of the interaction of a small molecule with intrinsically disordered p27^Kip1^ found little evidence of ligand-induced conformational space expansion [31]. Instead, ligand binding was shown to mainly shift the populations of pre-existing states. 

### 3.2. Inhibition of Aggregation by Induced Compaction

Dynamic interactions were also found to underlie the mechanism of α-synuclein aggregation inhibition by analogs of cyclized nordihydroguaiaretic acid (cNDGA) [125]. The structural basis of cNDGA inhibition was characterized using an array of biochemical and biophysical methods, including NMR and fluorescence correlation spectroscopy. The results revealed that cNDGA induced modest compaction of the conformational ensemble of monomeric α-synuclein, apparently mediated by dynamic and transient interactions with the protein and without hindering membrane association. cNDGA-treated α-synuclein is resistant to aggregation even when seeded with α-synuclein aggregates. Importantly, cNDGA was further shown to be effective in reducing α-synuclein-driven neurodegeneration in *C. elegans*. The observation that dynamic interactions between a small molecule and IDP could be functionally effective both in vitro and in vivo is very encouraging and supports the promise of targeting IDPs using dynamic interactions for therapeutics.

**Figure 3:**
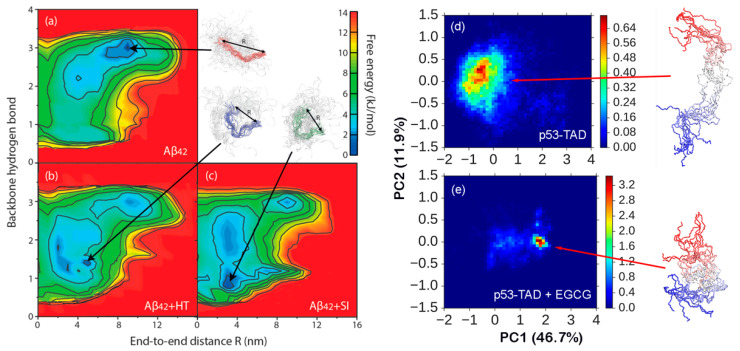
Conformational ensembles of Aβ42 with and without the ligands (**a**–**c**) and p53-TAD with and without ligands (**d**,**e**). The conformational space of Aβ42 is projected onto the number of backbone hydrogen bonds and end-to-end distance and that of p53-TAD is projected onto the first two principal components. The conformational ensembles were calculated using long timescale REST2 simulations in explicit solvent (10 and 1 μs per replica for Aβ42 and p53-TAD, respectively). Representative conformations are shown in backbone traces. This figure was adapted from [126,127]. See [126,127] for details on the simulation and analysis.

Induced compaction of the disordered conformational ensemble has also been predicted to underlie the inhibition mechanism of two drugs under clinical trials for treating Alzheimer’s diseases [126]. The disordered ensembles of Aβ_42_ with and without tramiprosate (homotaurine; HT) and *scyllo*-inositol (SI) were calculated using REST2 simulations in the CHARMM36m force field that lasted 10 μs per replica, making them the most extensive atomistic simulations of Aβ_42_ to date. The resulting ensembles are well converged and appear consistent with the NMR chemical shifts. Comparing the ensembles with and without the ligand showed that both HT and SI mainly reduced the β propensity in the C-terminal region with minimal secondary structure perturbation in the rest of the peptide. Intriguingly, both HT and SI were found to induce modest compaction of the conformational ensemble, particularly in the C-terminal segment that is known to be important for amyloid fibril formation (Figure 3a–c). Detailed analysis further revealed that the effects of both HT and SI binding were achieved via dynamic and nonspecific interactions with various backbone and sidechain moieties of the peptide. It is noteworthy that the conformational modulation effects of both HT and SI can be very difficult to detect at the ensemble level using bulk measurements, highlighting a critical role for reliable atomistic simulations that leverage recent advances in both protein force field quality and sampling capability. Nonetheless, additional validation is needed to support the predicted conformational shifts induced by drugs and to establish the roles of such conformational changes in the mechanisms of drug action. 

### 3.3. Modulating Regulatory IDPs via Dynamic Interactions

De novo atomistic simulations have also been integrated with NMR and biophysical experiments to examine how an anticancer drug, epigallocatechin gallate (EGCG), modulates the disordered unbound state of p53-TAD [127]. EGCG is a major active ingredient of green tea and has been reported to have anticancer effects in both animal studies and clinical trials [128,129,130]. The results suggested that EGCG also interacted dynamically with p53-TAD through numerous transient and nonspecific interactions, which appeared consistent with NMR chemical shift titration results. Multiple hydrophobic and particularly aromatic sidechains contribute significantly to EGCG binding. The dynamic interaction with EGCG was predicted to induce significant conformational compaction of p53-TAD in the N-terminal region (Figure 3d,e), which appears consistent with the SAXS measurements. The compaction could shield the p53-TAD site required for interacting with MDM2 and thus inhibit p53 degradation to promote its anticancer activities. It is noteworthy that EGCG has also been shown to be active in inhibiting the aggregation of multiple proteins, including Aβ peptides and α-synuclein [131,132]. The implication is that dynamic interactions of EGCG could provide a molecular basis for promiscuous selectivity, which is a fascinating property to investigate further using a combination of experiments and simulation. Recently, Neira et al. screened a set of FDA-approved drugs and identified 15 compounds that could bind NUPR1, a multi-functional IDP involved in pancreatic ductal adenocarcinoma [133,134]. NMR chemical shift analysis suggested that NUPR1 remained disordered in complex with all compounds, which is consistent with an inhibition mechanism involving transient and dynamic IDP–ligand interactions. Importantly, these compounds showed efficacy in cell-based assays, the most effective of which was found to completely arrest tumor growth in a mouse model.

## 4. Concluding Discussions

IDPs have remained an extremely challenging class of proteins to target using small molecules. Albeit limited, successful inhibitors have been discovered and designed for several IDPs involved in cancers and neurodegenerative diseases, suggesting that IDPs are not undruggable. Nonetheless, the unstructured and dynamic nature of IDPs is distinct from typical protein targets with well-defined binding pockets. It requires new conceptional frameworks to guide the development of novel strategies for discovering and designing small molecules that can modulate IDP functions. Traditional structure-based screening and lead optimization strategies are clearly inadequate, even though some success has been demonstrated in deploying existing tools to identify possible binding pockets and target pre-existing structural elements. Such structural elements are lightly populated and generally too small to harbor significant pockets for small molecular binding that relies on specific interactions to achieve high affinity. In fact, there is a great uncertainty on whether high-affinity binding to IDPs is feasible with small molecules (e.g., to meet the typical industrial standard of dissociation constants of nM or lower). It is encouraging that examples are emerging that small molecules could modulate the IDP ensembles entirely through dynamic nonspecific interactions. Importantly, there is evidence that high-affinity binding may not be necessary to induce functional responses in vitro and in vivo. This may reflect a fundamental nature of how IDPs mediate function in biology, in that the disordered ensemble of an IDP is poised to respond sensitively to a wide array of cellular signals to support signal transduction and cellular regulation [10]. Therefore, there is a great potential and promise for targeting IDPs through dynamic interactions with small molecules. 

It is noteworthy that IDPs have been found to play central roles in mediating liquid–liquid phase separation (LLPS) that underlies a range of cellular processes [135,136,137]. How small molecules may modulate the equilibrium and properties of these biological condensates is essentially unknown at this point. However, it is conceivable that LLPS may also be ideally targeted using dynamic interactions with small molecules that modulate the conformational flexibility and preference of disordered regions, which in turn modify the multivalent interaction profiles and entropic contribution that affect the condensation process as well as the properties of the condensate itself. 

Elucidating the molecular details of dynamic interactions between IDPs and small molecules will require further development and integration of new experimental and computational methodologies. The capability for reliable disordered ensemble characterization with and without small molecules will almost certainly be required for any future design and optimization strategy to discover drugs that target IDPs through dynamic interactions. This unfortunately remains a formidable task. Bulk experimental measurements on average properties alone are not sufficient to uniquely define the disordered ensemble. The dynamic and transient nature of molecular contacts can be very difficult to detect and resolve experimentally [138,139]. Leveraging significant recent advances in the protein force field quality, sampling techniques, and GPU computing, de novo atomistic simulations are now poised to help meet these challenges and play a pivotal role in establishing the molecular basis of dynamic IDP–small molecule interactions.
